# Pancreatogenic Type 3c Diabetes After Major Pancreatic Resections for Chronic Pancreatitis: A Single-Center Experience of More than 100 Surgical Cases

**DOI:** 10.3390/jcm14165817

**Published:** 2025-08-17

**Authors:** Dhruv J. Patel, Alexandra D. Nelson, Melissa E. Chen, Morgan S. Jones, Chirag S. Desai

**Affiliations:** 1 Department of Surgery, School of Medicine, Chapel Hill, NC 27599, USA; dhruvpatel1067@gmail.com; 2 School of Medicine, University of North Carolina, Chapel Hill, NC 27599, USA; alexandra_nelson@med.unc.edu; 3 Division of Abdominal Transplantation, Department of Surgery, School of Medicine, University of North Carolina, Chapel Hill, NC 27599, USA; melissa_chen@med.unc.edu; 4 Division of Endocrinology and Metabolism, Department of Medicine, School of Medicine, University of North Carolina, Chapel Hill, NC 27599, USA; morgan_jones@med.unc.edu; 5 Department of Surgery, School of Medicine, University of North Carolina, 4001 Burnett Womack Building, Chapel Hill, NC 27599, USA

**Keywords:** chronic pancreatitis, pancreatic surgery, TPIAT

## Abstract

**Background/Objectives:** The impact of surgical resection for chronic pancreatitis on subsequent endocrine outcomes remains unclear. **Methods:** A single-center analysis of patients with chronic pancreatitis who underwent either a parenchymal-preserving surgery (PPS) or a total pancreatectomy (TP) with/without islet autotransplantation (IAT) between 2018 and 2024 was performed. Preoperative and postoperative changes in hemoglobin A1C (HbA1C) and long-acting insulin dose were compared. Univariate and multivariate analysis was performed to identify factors associated with 1-year insulin independence. **Results**: A total of 104 patients underwent surgery for chronic pancreatitis between 2018 and 2024. A total of 35 (33.7%) patients underwent TPIAT, 8 (7.7%) underwent TP, and 61 (58.7%) underwent PPS (n = 18 Whipple, n = 38 distal pancreatectomy, n = 5 drainage procedure). Median HbA1C increased after surgery (5.7% vs. 6.8%, *p* < 0.001). The majority of patients (n = 73, 70.2%) were discharged postoperatively without any basal insulin requirement. Of the 31 patients discharged on basal insulin, 18 patients (58.1%) were not on basal insulin preoperatively; the other 13 patients (41.9%) that were on basal insulin preoperatively had a median change in their postoperative basal insulin dose of −5 units [IQR: −12–−1]. A total of 46 patients (52.3%) were insulin independent at one year, with PPS more favorable than TPIAT (47.6% vs. 21.7%, *p* < 0.001) and less likely to have been on preoperative basal insulin. **Conclusions:** Surgery for chronic pancreatitis resulted in an increase in HbA1C postoperatively; however, diabetes remained well-controlled as the majority of patients remained off basal insulin at one year from surgery. PPS patients were more likely to be insulin-independent.

## 1. Introduction

The sequalae of chronic pancreatitis include both progressive exocrine and endocrine pancreatic insufficiency, eventually resulting in pancreatogenic or Type 3c diabetes. Half of all patients with chronic pancreatitis will require surgical management, most commonly for intractable pain, and there is increasing evidence of beneficial outcomes from earlier operative intervention [1]. Surgical options include pancreatoduodenectomy (Whipple), body and tail resection (distal pancreatectomy, or ductal drainage procedures, collectively referred to as parenchymal-preserving surgery (PPS)). Operations involving resection of the entire pancreas include total pancreatectomy without autologous islet cell transplant (TP), and total pancreatectomy with autologous islet cell transplant (TPIAT) [2]. While surgery for chronic pancreatitis can result in improvement in pain and subsequent quality of life, it can have varying effects on endocrine outcomes. It is intuitive, and supported by the limited data, that postoperative quality of life can be dependent on the resolution or progression of Type 3c diabetes [3]. Longer follow-up data on postoperative survival have identified diabetic-related complications as a common cause of death, especially early mortality [4].

Patients undergoing pancreatic resection for oncologic purposes have a varied quality of pancreatic gland, resulting in altogether different pathophysiological changes in patients from the remanent pancreas. Patients with chronic pancreatitis are unique in that the endocrine function of their pancreata is already affected preoperatively. Islets of Langerhans can either be stressed or damaged in these patients’ pancreata. If they are stressed, surgical intervention to prevent ongoing inflammatory damage or removal of the diseased pancreas may have beneficial effects on endocrine outcomes, and, hypothetically, diabetes may be better controlled in these patients. There is good data about the onset or progression of diabetes upon pancreatic resection in cancer patients undergoing Whipple procedure or distal pancreatectomy; however, there is limited data in larger cohorts with chronic pancreatitis [5,6]. Hence, in order to describe the impact of surgery for chronic pancreatitis on Type 3c diabetes outcomes, we aim to compare preoperative versus postoperative glycemic control and insulin dependency after both PPS, TP, and TPIAT operations for chronic pancreatitis.

## 2. Materials and Methods

### 2.1. Patient Selection and Variables of Interest

All patients undergoing surgical treatment for chronic pancreatitis at a single, dedicated chronic pancreatitis and islet cell autotransplantation program at a tertiary care institution between 2018 and 2024 were retrospectively evaluated. Patients were included if they underwent either a parenchymal-preserving surgery (PPS), total pancreatectomy without autologous islet cell transplant (TP), or a total pancreatectomy with autologous islet cell transplant (TPIAT). PPS encompassed distal pancreatectomy (DP), Whipple, and drainage procedures. We included drainage procedures within PPS as parenchyma is preserved in these procedures, and these procedures have previously been found to be associated with pancreatogenic diabetes [7]. Evaluation prior to TPIAT was performed by a multidisciplinary committee with extensive investigation as described previously [8]. Endocrine functions were broadly evaluated with hemoglobin A1c (HbA1C), stimulated C-peptide, and continuous glucose monitoring (CGM) when feasible. All operations were performed by a single surgeon, and all TPIAT were performed with an islet cell isolation and infusion technique described previously by Desai et al. [9]. Patients had scheduled postoperative follow-up at six weeks, three months, six months, nine months, and one year after surgery. Patients were evaluated postoperatively by an endocrinology team that managed glucose levels. Patients were initiated on basal insulin if fasting glucose was above 180 mg/dL on two or more fasting levels prior to discharge. Prandial insulin was started if pre-meal glucose levels at lunch or dinner time were greater than 200 mg/dL. Among these general guidelines, there remained variability due to a large glucose management team. In the outpatient setting, patients had follow-up offered with an endocrinologist who specialized in pancreatogenic diabetes, yet many patients often returned to their community endocrinologist or primary care provider for follow-up. The decision to initiate or continue basal insulin was made on a case-by-case basis with no specific protocols for titration.

Demographic and clinical data of interest included age, sex, race, BMI, chronic pancreatitis etiology, and surgery type. Endocrine outcomes were tracked before and after surgery. Glycemic control was evaluated with HbA1c and CGM data. Preoperative diabetes was defined by a hemoglobin A1c ≥ 6.5% or a basal insulin requirement. The most recent HbA1c prior to surgery was compared to the lowest HbA1c at least three months after surgery and within one postoperative year. The American Diabetes Association recommends a HbA1C < 7% without significant hypoglycemia as a general recommendation for adequate glycemic control in diabetic patients, so this guideline was taken into consideration along with the amount of basal insulin required to achieve a HbA1C < 7% in order to contextualize any potential elevations in postoperative A1C [10]. CGM data was inconsistently available, and when it was available, the percentage of time in the hypoglycemia range (less than 70 mg/dL) was reviewed before surgery and compared to the lowest value within one postoperative year. In addition to CGM data, we defined clinically significant hyperglycemia as glucose levels less than 54 mg/dL requiring assistance for treatment. Insulin independence at one postoperative year was defined as no usage of basal insulin. Due to significant variation in the setting of fluctuating oral intake postoperatively, bolus or prandial insulin was often sporadic and inconsistent. Basal insulin requirements were compared based on the patient’s dosage on the day of surgery, the day of postoperative discharge, and the dosage on the closest ambulatory visit one year after surgery. The change in basal insulin dosage after surgery was evaluated for those patients who were on insulin preoperatively. For the TPIAT cohort, preoperative-stimulated C-peptide levels were collected, as well as the islet cell yield (IEQ/kg). An optimal islet cell yield was defined as at least 2500 IEQ/kg transplanted. The study was conducted in accordance with the Declaration of Helsinki and approved by the Institutional Review Board of the University of North Carolina School of Medicine (protocol code 19-2591 and date of approval 30 September 2019). Informed consent was waived by the institutional review board since surgical consent incorporates a research component.

### 2.2. Statistical Analysis

Statistical analysis was performed using RStudio statistical software (R Foundation for Statistical Computing, Vienna, Austria, version 4.4.1). All tests were two-sided, and a *p* value of < 0.05 was our threshold for statistical significance. The Wilcoxon signed-rank test was used to compare preoperative and postoperative HbA1c. Univariate analysis using Pearson’s chi-squared test, Fisher’s exact test, and the Mann–Whitney U test was performed to evaluate factors associated with surgery type and insulin independence one year after surgery. A multivariate analysis was then performed to identify adjusted factors associated with insulin independence, incorporating variables that were statistically significant on univariate analysis and clinically relevant. Moreover, as we anticipated that patients who underwent a TP would all be on basal insulin on postoperative follow-up, we excluded patients who underwent a TP from our multivariable model, and we grouped all PPS as our reference for surgery type in order to assess the adjusted probability of insulin independence among the TPIAT cohort. We additionally performed a univariate subgroup analysis of the TPIAT cohort to more specifically identify predictors of insulin independence among that cohort.

## 3. Results

### 3.1. Baseline Characteristics and Endocrine Outcomes for Total Cohort

Between 2018 and 2024, 104 patients underwent surgery for chronic pancreatitis at our center. A total of 61 (58.7%) patients underwent PPS (n = 18 Whipple, n = 38 distal pancreatectomy, n = 5 drainage procedure); 43 (41.3%) patients underwent TP with (n = 35) or without IAT (n = 8) [Table 1]. The M:F was 50:50, and the majority of the cohort (75.0%) was white. The two most common etiologies for chronic pancreatitis were idiopathic (26.9%) and alcohol (25.0%).

The majority of patients in our study for which one-year follow-up was available had follow-up for both HbA1C and insulin independence (88/91, 96.7%). Three patients were lost to follow-up regarding one-year insulin independence data, and of these three patients, two underwent a distal pancreatectomy and one underwent a Whipple. There was a statistically significant increase in the median HbA1C after surgery (5.7% [IQR: 5.3–6.8] vs. 6.8% [5.9–7.9], *p* < 0.001). The majority (n = 87, 83.7%) of the cohort was not on basal insulin preoperatively, and the majority (n = 73, 70.2%) of the cohort remained off basal insulin on the day of postoperative discharge (Table 2). A total of 4 of the 73 patients (5.5%) who were discharged without long-acting insulin were on preoperative basal insulin with a mean dose of 10.5 units, signifying improvement in glycemic control. For the 31 patients who were discharged after surgery with basal insulin, 18 patients (58.1%) were not on basal insulin preoperatively, and those patients had a median postoperative basal requirement of 12 units [IQR: 8–14]. The 13 patients (41.9%) who were on basal insulin preoperatively had a median decrease in their postoperative basal insulin dose of 5 units [IQR: −12–−1]. Seven patients had CGM data from before surgery and for 1 complete year after surgery. The preoperative percentage of time in the hypoglycemia range for these patients was the following: 15%, 11.5%, 0%, 14%, 0%, 0%, and 66%. The lowest respective percentage of time spent in the hypoglycemia range within a 1-year follow-up after surgery was the following: 1%, 0%, 0%, 0%, 0%, 0%, and 0%. At one-year follow-up, there were no episodes of clinically significant hypoglycemia (glucose less than 54 mg/dL requiring treatment). Eighty-eight (84.6%) patients had 1-year follow-up data on insulin dependency, with the majority (n = 46, 52.3%) remaining independent from long-acting insulin. For the 42 (47.7%) patients who were on basal insulin at 1-year follow-up, the median dose was 14 units [IQR: 10–12]. A total of 15 of these 42 patients (35.7%) were initially discharged postoperatively without any long-acting insulin.

### 3.2. Univariate Analysis for Factors Associated with Surgery Type and 1-Year Insulin Independence

On univariate analysis by surgery type, patients undergoing TP as well as TPIAT were younger than patients undergoing DP, Whipple, or drainage procedures (*p* < 0.001) [Table 3]. Patients undergoing TPIAT were also more likely to have a hereditary etiology for chronic pancreatitis and less likely to have chronic pancreatitis secondary to alcohol. With respect to preoperative HbA1C and preoperative insulin use, patients who underwent TP were more likely to have a higher A1C and were more likely to be on preoperative insulin compared to all other surgery types. All patients who underwent a TP required insulin at one-year postoperative follow-up.

In assessing the entire cohort for factors associated with 1-year insulin independence, we found that patients who were insulin independent had a lower preoperative HbA1C (5.5 vs. 6.7, *p* < 0.001) and were less likely to be on preoperative basal insulin (2.2% vs. 33.3%, *p* < 0.001) [Table 4]. They were also less likely to have undergone TPIAT (21.7% vs. 47.6%, *p* < 0.001).

### 3.3. Multivariate Analysis of Adjusted Predictors of 1-Year Insulin Independence

To identify adjusted factors associated with one-year insulin independence, a multivariate regression was performed, controlling for age, preoperative HbA1C, preoperative insulin use, and surgery type. On adjusted analysis, preoperative insulin was no longer associated with insulin independence [Adjusted Odds Ratio (aOR): 0.49 (0.02–6.38), *p* = 0.6202] while increasing preoperative HbA1C remained associated with decreased likelihood for insulin independence [aOR: 0.27 (0.08–0.70), *p* = 0.016] [Table 5]. TPIAT, compared to PPS, remained a predictor of insulin independence [aOR: 0.08 (0.01–0.34), *p* = 0.002] at one postoperative year.

### 3.4. Subgroup Analysis of TPIAT

Since 2018, 35 total cases of TPIAT have been performed. The 1-year insulin independence data was available for 30 of 35 cases, and of the 30 patients who underwent TPIAT, 10 (33.3%) remained off long-acting insulin at 1-year follow-up. On univariate analysis examining factors associated with 1-year insulin independence among the TPIAT subgroup, there was no difference in preoperative HbA1C (5.8% vs. 5.5%, *p* = 0.185), preoperative-stimulated C-peptide level (5.0 vs. 4.8, *p* = 0.582), or preoperative basal insulin requirement (10% vs, 10%, *p* = 1.000) between patients who did and did not require basal insulin at 1-year follow-up [Table 6]. There appeared to be trends towards higher islet cell yield [4341.8 IQR: (3242.4–5339.3) vs. 1804.9 IQR: (1044.0–4037.1), *p* = 0.078] and higher percentage of optimal islet cell threshold of at least 2500 IEQ/kg (80% vs. 40%, *p* = 0.058) among patients who were insulin independent at 1-year; however, these trends were not statistically significant.

## 4. Discussion

The endocrine results of our total cohort of chronic pancreatitis operations are comparable to prior studies examining the impact of pancreatic surgery on glycemic control and insulin requirements. Patients who underwent PPS were more likely to remain insulin independent at 1 postoperative year compared to those who underwent TPIAT. One of the noteworthy findings from our study is that for the select group of patients on insulin preoperatively who were then discharged on insulin, those patients had a reduction in their basal insulin requirement at the time of discharge. A recent meta-analysis of 82 studies of patients who had undergone a partial pancreatectomy for both benign and malignant indications found the resolution rate of pre-existing diabetes to be 25.8% after surgery in a cohort of 1233 patients [11]. In a more selective single-institution, retrospective review of 224 patients undergoing surgery for chronic pancreatitis, 7% of a cohort of 68 patients with preoperative diabetes experienced resolution of their diabetes [12]. Another study examining changes in glycemic control before and after the Whipple procedure in 861 patients with diabetes found comparable rates of diabetic resolution in those undergoing surgery for benign or malignant indications (20.1% vs. 20.5%, respectively) [5]. We postulate that diabetes is better controlled after surgery for chronic pancreatitis, as the source of the proinflammatory stress of chronic pancreatitis is eliminated. The aforementioned study also found that preoperative use of insulin was associated with lower rates of improvement of diabetes [odds ratio (OR): 0.27, 95% CI (0.17–0.41), *p* < 0.001], after adjusting for periampullary cancer and chronic pancreatitis. Our analysis was performed on a cohort of patients who all had a clinical diagnosis of chronic pancreatitis, and while we initially found preoperative basal insulin use to be associated with a decreased likelihood of 1-year insulin independence after surgery, this association was no longer significant after controlling for age, preoperative HbA1C, and surgery type.

Prior studies assessing the impact of surgery type on endocrine outcomes after surgery have found distal pancreatectomy to be associated with a higher probability for the development of new-onset diabetes. The most common surgery performed in our analysis was a distal pancreatectomy (36.5%), and the majority of patients who underwent a distal pancreatectomy were independent of insulin at 1-year follow-up (65.5%). In their meta-analysis, Wei et al. reported a 23.7% incidence of de novo diabetes after distal pancreatectomy in a cohort of 3162 patients [11]. Malka et al. reported an association between distal pancreatectomy and both diabetes [(OR 2.4, 95% CI (1.6–3.8), *p* < 0.001] and insulin requirement [(OR 2.6, 95% CI (1.4–4.7), *p* = 0.001] on multivariable analysis in their single-institution series comprising 231 patients who underwent surgery for chronic pancreatitis [13]. A series reporting a 20-year follow-up of 77 patients who underwent distal pancreatectomy for chronic pancreatitis found a 23% diabetic morbidity rate related to surgery [14]. The surgical rationale for an increased likelihood of developing endocrine insufficiency after a distal pancreatectomy may be related to the heterogenous distribution of islet cells along the pancreas, with a higher density of islet cells found in the distal pancreas [15]. However, there may also be a physiologic contribution from recurrent chronic pancreatitis after a distal pancreatectomy that may worsen Type 3c diabetes.

About one-third of our cohort underwent a TPIAT, and endocrine outcomes for those who underwent TPIAT in our study are equivalent to prior analyses. The one-year insulin independence rate for patients who underwent TPIAT in our study was 33.3%. Darden et al. reported a 28.2% one-year insulin independence rate in their cohort of 131 TPIAT cases [16]. Morgan et al. and Nanno et al. reported rates of insulin independence of 29% and 33% at one-year follow-up after TPIAT, respectively [17,18]. The prevalence (10%) of preoperative long-acting insulin use among our cohort of TPIAT patients with one-year follow-up was limited; therefore, unlike the analysis for our total cohort, we did not observe an association between preoperative insulin use and one-year insulin independence. A trend towards an association between preoperative diabetes and a reduced likelihood of one-year insulin independence has been previously observed. In a 45-patient series, Haddad et al. reported that none of the seven patients with diabetes preoperatively were insulin independent one year after TPIAT [19]. Bachul et al. reported that none of the 5 patients with diabetes preoperatively in their 34-patient cohort were insulin independent at one-year follow-up [20]. One of the predictors that has previously been shown to portend outcomes is the islet cell yield [17,20]. While not statistically significant, there was a trend towards a higher islet cell yield among the subgroup of patients who were insulin independent after TPIAT in our study. Moreover, prior studies have suggested an optimal islet yield threshold of at least 2500 IEQ/kg in order to achieve one-year insulin independence [18,21]. Again, while not statistically significant, eight of the ten patients (80%) who were insulin independent at one-year received an islet yield of at least 2500 IEQ/kg; only eight of the twenty patients (40%) who were on long-acting insulin at one-year follow-up obtained an islet yield of at least 2500 IEQ/kg. It is also worthy of discussion that none of the patients who received a TPIAT in our study suffered from hypoglycemic events after surgery, which is a metric of brittle diabetes not always captured in prior cohort studies on TPIAT.

There are several limitations of our analysis. First, the retrospective design of our study is subject to selection bias. Our analysis, especially for the TPIAT cohort, is underpowered due to the small sample size. Therefore, our results should be applied with caution for patients who undergo TPIAT. All cases were performed by a single surgeon because of the nature of the complexity of the operations and experience required, which may make an argument for limiting the generalizability of our findings. However, it can be argued as well that the endocrine outcomes of this analysis are physiological outcomes rather than surgical. Our definition of insulin independence has some limitations, as we encountered some challenges with accurately capturing the usage of prandial or bolus insulin. In addition to insulin independence, it would be of interest to assess data on the percentage of time in the hypoglycemia range after surgery, though we only report this metric for seven patients who underwent TPIAT. Insulin independence is commonly reported when evaluating endocrine outcomes after TPIAT; however, there may be other markers of glycemic control perioperatively that may better assess glucose intolerance and islet function. There may be a role for an oral glucose tolerance test (OGTT) or a homeostatic model assessment of beta cell function (HOMA-B) in preoperative and postoperative evaluation of endocrine function [22]. We additionally report a short follow-up time, yet there is evidence to suggest that the median time to onset of diabetes after pancreatectomy is typically within one year of surgery [23].

In conclusion, we present a series of patients at our institution who underwent PPS, TP, and TPIAT operations for chronic pancreatitis, and having these groups combined is one of the strengths of this analysis. We found surgery for chronic pancreatitis to result in good endocrine outcomes, for no patients in our analysis suffered from postoperative hypoglycemic events, and the majority remained free from insulin at one-year follow-up. Diseased pancreas as a sequalae of chronic pancreatitis may generate a stress response manifesting with hyperglycemia that requires exogenous insulin, and the removal of diseased pancreas may in fact improve glycemic control within this population of patients, eliminating the need for exogenous insulin. Future studies can further elucidate the impact of surgery on Type 3c diabetes outcomes by assessing multiple measures of glycemic control, providing a larger analysis of TPIAT, and tracking endocrine outcomes over longer follow-up times with patients from multiple centers.

## Figures and Tables

**Table 1:** Demographic and clinical characteristics for patients undergoing surgery for chronic pancreatitis.

Variable	N = 104 (%)
Age, median (IQR)	47.0 (37–59)
Sex	
Male	52 (50%)
Female	52 (50%)
Race	
White	78 (75.0%)
Black	15 (14.4%)
Asian	1 (1.0%)
Other (Hispanic)	9 (8.7%)
BMI, median IQR	26 (23.1–30.3)
Chronic Pancreatitis Etiology	
Idiopathic	28 (26.9%)
Alcohol	26 (25.0%)
Hereditary/Genetic	16 (15.4%)
Gallstone/Obstruction	14 (13.5%)
Divisum	10 (9.6%)
Autoimmune	4 (3.8%)
Other	6 (5.8%)
Surgery	
Distal Pancreatectomy	38 (36.5%)
TPIAT	35 (33.7%)
Whipple	18 (17.3%)
Total	8 (7.7%)
Drainage	5 (4.8%)

**Table 2:** Glycemic control and insulin requirements before and after surgery for chronic pancreatitis.

Variable	Overall
Preoperative HbA1C (%), median (IQR)	5.7 (5.3–6.8)
Postoperative HbA1C (%), median (IQR)	6.8 (5.9–7.9)
Preoperative Basal Insulin	
No	87 (83.7%)
Yes	17 (16.3%)
Postoperative Discharge Basal Insulin	
No	73 (70.2%)
Yes	31 (29.8%)
Postoperative 1-Year Insulin Independence	
No	42 (47.7%)
Yes	46 (52.3%)
Postoperative 1-Year Antihyperglycemic Use	
No	69 (78.4%)
Yes	19 (21.6%)

**Table 3:** Univariate analysis of factors associated with surgery type.

Variable	DP	Drainage	Whipple	TP	TPIAT	*p* Value
	N = 38	N = 5	N = 18	N = 8	N = 35	
Age, median (IQR)	48 (38.2–62.8)	58 (44–59)	56 (47–63.5)	46.5 (42.2–48.2)	40 (31.5–49)	0.001
Sex						0.074
Female	21 (55.3)	1 (20.0)	6 (33.3)	2 (25.0)	22 (62.9)	
Male	17 (44.7)	4 (80.0)	12 (66.7)	6 (75.0)	13 (37.1)	
Race						0.166
White	23 (62.2)	5 (100.0)	16 (88.8)	4 (50.0)	30 (85.7)	
Black	10 (27.0)	0 (0.0)	1 (5.6)	2 (25.0)	2 (5.7)	
Asian	0 (0.0)	0 (0.0)	0 (0.0)	0 (0.0)	1 (2.9)	
Other (Hispanic)	4 (10.8)	0 (0.0)	1 (5.6)	2 (25.0)	2 (5.7)	
BMI, median (IQR)	27.2 (24.2–32.2)	24.7 (24.7–25.9)	27 (22.2–29.7)	23.3 (21.1–25.7)	24.6 (22.8–29.7)	0.179
Chronic Pancreatitis Etiology						0.006
Alcohol	8 (21.1)	2 (40.0)	6 (33.3)	2 (25.0)	8 (22.9)	
Autoimmune	1 (2.6)	0 (0.0)	0 (0.0)	2 (25.0)	1 (2.9)	
Divisum	1 (2.6)	0 (0.0)	3 (16.7)	1 (12.5)	5 (14.3)	
Hereditary	2 (5.3)	0 (0.0)	1 (5.6)	1 (12.5)	12 (34.3)	
Gallstone/Obstruction	8 (21.1)	2 (40.0)	2 (11.1)	2 (25.0)	0 (0.0)	
Idiopathic	14 (36.8)	1 (20.0)	5 (27.8)	0 (0.0)	8 (22.9)	
Other	4 (10.5)	0 (0.0)	1 (5.6)	0 (0.0)	1 (2.9)	
Preoperative Insulin Use						<0.001
No	33 (86.8)	4 (80.0)	17 (94.4)	2 (25.0)	31 (88.6)	
Yes	5 (13.2)	1 (20.0)	1 (5.6)	6 (75.0)	4 (11.4)	
Preoperative HbA1C (%), median (IQR)	5.5 (5.2–6.7)	6.9 (6–8.3)	5.4 (5.2–5.7)	8.1 (6.7–8.3)	5.6 (5.3–6.2)	0.034
One-Year Oral Antihyperglycemic Use						0.086
No	23 (79.3)	3 (60.0)	15 (93.8)	8 (100.0)	20 (66.7)	
Yes	6 (20.7)	2 (40.0)	1 (6.2)	0 (0.0)	10 (33.3)	
One-Year Insulin Independence						<0.001
No	10 (34.5)	1 (20.0)	3 (18.8)	8 (100.0)	20 (66.7)	
Yes	19 (65.5)	4 (80.0)	13 (81.2)	0 (0.0)	10 (33.3)	

**Table 4:** Univariate analysis of factors associated with one-year insulin independence following surgery for chronic pancreatitis.

Variable	Insulin Independence		*p* Value
	No	Yes	
Age, Median (IQR)	48.0 (39.0–58.0)	45.5 (37.0–59.8)	0.776
Sex			
Female	18 (42.9)	24 (52.2)	0.509
Male	24 (57.1)	22 (47.8)	
Race			
White	31 (73.8)	39 (84.8)	0.429
Black	7 (16.7)	4 (8.7)	
Asian	1 (2.4)	0 (0.0)	
Other (Hispanic)	3 (7.1)	3 (6.5)	
BMI, median (IQR)	26.8 (23.0–30.0)	24.6 (23.0–29.9)	0.528
Chronic Pancreatitis Etiology			0.228
Alcohol	10 (23.8)	13 (28.3)	
Autoimmune	3 (7.1)	0 (0.0%)	
Divisum	6 (14.3)	3 (6.5%)	
Hereditary	6 (14.3%)	6 (13.0%)	
Gallstone/Obstruction	7 (16.7%)	7 (15.2)	
Idiopathic	10 (23.8)	13 (28.3)	
Other	0 (0.0%)	4 (8.7%)	
Surgery			
DP	10 (23.8)	19 (41.3)	<0.001
Whipple	3 (7.1)	13 (28.3)	
Drainage	1 (2.4)	4 (8.7)	
TP	8 (19.0)	0 (0.0%)	
TPIAT	20 (47.6)	10 (21.7%)	
Preoperative HbA1C (%), median (IQR)	6.7 (5.6–8.2)	5.5 (5.2–5.7)	<0.001
Preoperative Insulin			
No	28 (66.7)	45 (97.8)	<0.001
Yes	14 (33.3)	1 (2.2)	
One-Year Antihyperglycemic Use			
0	34 (81.0)	35 (76.1)	0.768
1	8 (19.0)	11 (23.9)	

**Table 5:** Multivariate analysis of adjusted predictors of one-year insulin independence following surgery for chronic pancreatitis.

Variable	Adjusted Odds Ratio (95% Confidence Interval)	*p* Value
Age	0.99 (0.94–1.05)	0.728
Preoperative Insulin Use	0.49 (0.02–6.38)	0.602
Preoperative HbA1C	0.27 (0.08–0.70)	0.016
TPIAT	0.08 (0.01–0.34)	0.002

**Table 6:** Univariate analysis of factors associated with one-year insulin independence following TPIAT.

Variable	Insulin Independence		*p* Value
	No	Yes	
Age, median (IQR)	39.5 (36.5–48.5)	43.0 (25.8–47.0)	0.860
Sex			
Female	12 (60.0)	8 (80.0)	0.387
Male	8 (40.0)	2 (20.0)	
Race			
White	17 (85.0)	10 (100.0)	1.000
Black	1 (5.0)	0 (0.0)	
Asian	1 (5.0)	0 (0.0)	
Other (Hispanic)	1 (5.0)	0 (0.0)	
BMI, median (IQR)	25.7 (22.9–29.6)	23.5 (22.1–24.4)	0.202
Chronic Pancreatitis Etiology			1.000
Alcohol	7 (35.0)	1 (10.0)	
Autoimmune	1 (5.0)	0 (0.0)	
Divisum	4 (20.0)	1 (10.0)	
Hereditary	5 (25.0)	4 (40.0)	
Gallstone/Obstruction	0 (0.0)	0 (0.0)	
Idiopathic	3 (15.0)	3 (30.0)	
Other	0 (0.0%)	1 (10.0)	
Preoperative HbA1C (%), median (IQR)	5.8 (5.4–6.8)	5.5 (5.2–5.7)	0.185
Preoperative-Stimulated C-peptide (%), median (IQR)	5.0 (3.8–6.3)	4.8 (4.1–5.8)	0.582
Preoperative Insulin			1.000
No	18 (90.0)	9 (90.0)	
Yes	2 (10.0)	1 (10.0)	
Islet Cell Yield (IEQ/kg), median (IQR)	1804 (1044–4.037.1)	4341.8 (3242.4–5339.3)	0.078
Optimal Islet Cell Yield (IEQ/kg)			
<2500	12 (60.0)	2 (20.0)	0.058
≥2500	8 (40.0)	8 (80.0)	
One-Year Antihyperglycemic Use			
0	15 (75.0)	5 (50.0)	0.231
1	5 (25.0)	5 (50.0)	

## Data Availability

The data source is available upon request.
